# Endobronchial ultrasound-guided transbronchial needle aspiration versus mediastinoscopy for mediastinal staging of lung cancer: A protocol for a systematic review of economic evaluation studies

**DOI:** 10.1097/MD.0000000000017242

**Published:** 2019-09-27

**Authors:** João Pedro Steinhauser Motta, José Roberto Lapa e Silva, Caroliny Samary Lobato, Vanessa Souza Mendonça, Ricardo E. Steffen

**Affiliations:** a Universidade Federal do Rio de Janeiro, Instituto de Doenças do Tórax; b Universidade Federal do Rio de Janeiro; c Universidade Estadual do Rio de Janeiro, Instituto de Medicina Social, Rio de Janeiro, Brazil.

**Keywords:** cost-effectiveness, EBUS, mediastinoscopy, systematic review

## Abstract

**Background:**

Lung cancer is a major health problem, with estimates of 1.6 million tumor-related deaths annually worldwide. The emergence of endobronchial ultrasound (EBUS), a minimally invasive procedure capable of providing valuable information for primary tumor diagnosis and mediastinal staging, significantly changed the approach of pulmonary cancer, becoming part of the routine mediastinal evaluation of lung cancer in developed countries. Some economic evaluation studies published in the last 10 years have already analyzed the incorporation of the EBUS technique in different health systems. The aim of this systematic review is to synthesize the relevant information brought by these studies to better understand the economic effect of the implementation of this staging tool.

**Methods:**

The systematic review will be reported using the Preferred Reporting Items for Systematic Reviews and Meta-Analyses (PRISMA) statement guidelines. Eletronic databases (Medline, Lilacs, Embase, Cochrane Library of Trials, Web of Science, Scopus, National Health System Economic Evaluation Database) will be searched for full economic analyses regarding the use of EBUS-guided transbronchial needle aspiration (EBUS-TBNA) compared to the surgical technique of mediastinoscopy for the mediastinal staging of lung cancer. Two authors will perform the selection of studies, data extraction, and the assessment of risk of bias. Occasionally, a senior reviewer will participate, if necessary, on study selection or data extraction.

**Results:**

Results will be published in a peer-reviewed journal.

**Conclusion:**

This review may influence a more cost-effective mediastinal staging approach for patients with lung cancer around the world and help health decision makers decide whether the EBUS-TBNA technique should be incorporated into their health systems and how to do it efficiently.

**Protocol Registry:**

PROSPERO 42019107901.

## 1 Introduction

Lung cancer is a major health problem, with estimates of 1.6 million tumor-related deaths annually worldwide.^[1]^ With the exception of a small proportion of patients diagnosed at an early stage of the disease or others with known distant metastasis, the majority of patients with lung cancer will have the indication of an invasive staging of the mediastinum.^[2]^ The emergence of endobronchial ultrasound (EBUS), a minimally invasive procedure capable of providing valuable information for primary tumor diagnosis and mediastinal staging, significantly changed the approach of pulmonary cancer, becoming part of the routine mediastinal evaluation of lung cancer in developed countries.^[3,4]^ A recent systematic review and meta-analysis of randomized controlled trials and observational studies comparing EBUS with mediastinoscopy suggested an equivalence of the 2 procedures for mediastinal staging of lung cancer, with a lower complication rate favoring the endosonographic approach.^[5]^ As a new method being incorporated by different health systems, the use of EBUS may lead to a shift in clinical and cost outcomes. Some economic evaluation studies published in the last 10 years have already analyzed the incorporation of the EBUS technique in different health systems.^[6–8]^ The aim of this systematic review is to synthesize the relevant information brought by these studies to better understand the economic effect of the implementation of this staging tool.

## 2 Methods

This systematic review will be reported using the Preferred Reporting Items for Systematic Reviews and Meta-Analyses (PRISMA) statement guidelines.^[9]^ The protocol of this systematic review has been registered on the International Prospective Register of Systematic Reviews (PROSPERO), registry number **CRD42019107901**.

### 2.1 Research problem

The PICO strategy was used to formulate the research problem (Table 1).

**Table 1:**
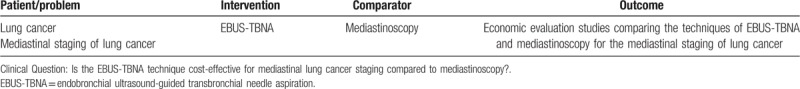
PICO strategy used to formulate the research Problem.

### 2.2 Search strategy

The literature search will be divided into 3 parts:

1. Search for systematic reviews on progress or already published about this review topic on PROSPERO.
2. Search in electronic databases: MEDLINE (via Pubmed), EMBASE, LILACS, Cochrane Library of Trials, Web of Science, Scopus, National Health System Economic Evaluation Database (NHS EED).
3. Cross-analysis of references of selected articles in the systematic review.

Search keys were constructed considering descriptors of terms related to the disease (lung cancer/mediastinal staging of lung cancer) and the technologies of interest (EBUS-transbronchial needle aspiration (TBNA) and mediastinoscopy) combined with a specific filter for economic studies (search filter developed by the Canadian Agency for Drugs and Technologies in Health).^[10]^ The search will be conducted on Medline and adapted for the other databases, according to the following search strategy: ((((“Lung Neoplasms”[Title/Abstract] OR “Pulmonary Neoplasms”[Title/Abstract] OR “Lung Neoplasm”[Title/Abstract] OR “Neoplasm, Lung”[Title/Abstract] OR “Pulmonary Neoplasm”[Title/Abstract] OR “Lung Cancer”[Title/Abstract] OR “Cancer, Lung”[Title/Abstract] OR “Lung Cancers”[Title/Abstract] OR “Pulmonary Cancer”[Title/Abstract] OR “Pulmonary Cancers”[Title/Abstract] OR “Cancer of the Lung”[Title/Abstract] OR “Cancer of Lung”[Title/Abstract] OR “Tumor Staging”[Title/Abstract] OR “Cancer Staging”[Title/Abstract] OR “TNM Staging” [Title/Abstract] OR “TNM Staging System”[Title/Abstract] OR “TNM Staging Systems” [Title/Abstract] OR “TNM Classification”[Title/Abstract] OR “Classification, TNM” [Title/Abstract] OR “TNM Classifications”[Title/Abstract]) OR “Lung Neoplasms” [MeSH Terms]) AND (EBUS-TBNA[Title/Abstract] OR EBUS[Title/Abstract] OR “endobronchial ultrasound”[Title/Abstract] OR “endoscopic ultrasound” [Title/Abstract] OR “endobronchial ultrasonography”[Title/Abstract] OR “endosonography”[Title/Abstract] OR “endobronchial ultrasound-guided” [Title/Abstract] OR “transbronchial needle aspiration”[Title/Abstract] OR “fine needle aspiration” [Title/Abstract] OR “minimally invasive endoscopic staging”[Title/Abstract])) AND ((“mediastinoscopy”[MeSH Terms] OR “mediastinoscopy”[All Fields]) OR (“mediastinoscopy”[MeSH Terms] OR “mediastinoscopy”[All Fields] OR “mediastinoscopies”[All Fields]) OR (“mediastinoscopy”[MeSH Terms] OR “mediastinoscopy”[All Fields] OR (“mediastinoscopic”[All Fields] AND “surgical”[All Fields] AND “procedures”[All Fields])) OR (“mediastinoscopy”[MeSH Terms] OR “mediastinoscopy”[All Fields] OR (“mediastinoscopic”[All Fields] AND “surgical”[All Fields] AND “procedure”[All Fields])) OR (“mediastinoscopy”[MeSH Terms] OR “mediastinoscopy”[All Fields] OR (“procedure”[All Fields] AND “mediastinoscopic”[All Fields] AND “surgical”[All Fields])) OR (“mediastinoscopy”[MeSH Terms] OR “mediastinoscopy”[All Fields] OR (“procedures”[All Fields] AND “mediastinoscopic”[All Fields] AND “surgical”[All Fields])) OR (“mediastinoscopy”[MeSH Terms] OR “mediastinoscopy”[All Fields] OR (“surgical”[All Fields] AND “procedure”[All Fields] AND “mediastinoscopic”[All Fields])) OR (“mediastinoscopy”[MeSH Terms] OR “mediastinoscopy”[All Fields] OR (“surgery”[All Fields] AND “mediastinoscopic”[All Fields])) OR (“mediastinoscopy”[MeSH Terms] OR “mediastinoscopy”[All Fields] OR (“surgical”[All Fields] AND “procedures”[All Fields] AND “mediastinoscopic”[All Fields])) OR “Mediastinoscopic Surgery”[All Fields] OR (“mediastinoscopy”[MeSH Terms] OR “mediastinoscopy”[All Fields] OR (“mediastinoscopic”[All Fields] AND “surgeries”[All Fields])) OR (“mediastinoscopy”[MeSH Terms] OR “mediastinoscopy”[All Fields] OR (“surgeries”[All Fields] AND “mediastinoscopic”[All Fields])))) AND (“economics”[MeSH Terms:noexp] OR “Costs and Cost Analysis”[mh] OR “economics, nursing”[MeSH Terms] OR “economics, medical”[MeSH Terms] OR “economics, pharmaceutical”[MeSH Terms] OR “economics, hospital”[MeSH Terms] OR “economics, dental”[MeSH Terms] OR “budgets”[MeSH Terms] OR (budget[tiab] OR budget’[tiab] OR budget's[tiab] OR budgetable[tiab] OR budgetaire[tiab] OR budgetarily[tiab] OR budgetary[tiab] OR budgeted[tiab] OR budgeteering[tiab] OR budgeters[tiab] OR budgetfor[tiab] OR budgetierung[tiab] OR budgeting[tiab] OR budgeting’[tiab] OR budgeting's[tiab] OR budgetizing[tiab] OR budgetrestricted[tiab] OR budgetry[tiab] OR budgets[tiab] OR budgets’[tiab] OR budgetsis[tiab] OR budgett[tiab] OR budgett's[tiab] OR budgetting[tiab]) OR (economic[tiab] OR economic’[tiab] OR economic”[tiab] OR economic's[tiab] OR economica[tiab] OR economical[tiab] OR economical’[tiab] OR economicall[tiab] OR economically[tiab] OR economically’[tiab] OR economicallyoriented[tiab] OR economicallyuncovered[tiab] OR economicalness[tiab] OR economicaly[tiab] OR economicamente[tiab] OR economicas[tiab] OR economicbranches[tiab] OR economiche[tiab] OR economici[tiab] OR economicissue[tiab] OR economicist[tiab] OR economicist’[tiab] OR economicity[tiab] OR economiclly[tiab] OR economicmicro[tiab] OR economico[tiab] OR economicomathematical[tiab] OR economicos[tiab] OR economicosocial[tiab] OR economicperformance[tiab] OR economicpubguidelines[tiab] OR economics[tiab] OR economics’[tiab] OR economicsanalysis[tiab] OR economicscience[tiab] OR economicus[tiab] OR economicus’[tiab] OR economicus’rationalism[tiab] OR economicwise[tiab]) OR cost[tiab] OR costs[tiab] OR costly[tiab] OR costing[tiab] OR price[tiab] OR prices[tiab] OR pricing[tiab] OR (pharmacoeconomic[tiab] OR pharmacoeconomical[tiab] OR pharmacoeconomically[tiab] OR pharmacoeconomics[tiab]) OR (pharmaco economic[tiab] OR pharmaco economical[tiab] OR pharmaco economically[tiab] OR pharmaco economics[tiab]) OR expenditure[tiab] OR expenditures[tiab] OR expense[tiab] OR expenses[tiab] OR financial[tiab] OR finance[tiab] OR finances[tiab] OR financed[tiab] OR value for money[tiab] OR (monetary value[tiab] OR monetary values[tiab]) OR “models, economic”[MeSH Terms] OR (economic model[tiab] OR economic modeling[tiab] OR economic modelling[tiab] OR economic models[tiab]) OR “markov chains”[MeSH Terms] OR markov[tiab] OR “monte carlo method”[MeSH Terms] OR monte carlo[tiab] OR “decision theory”[MeSH Terms] OR (decision tree[tiab] OR decision treeboost[tiab] OR decision trees[tiab]) OR (decision analyses[tiab] OR decision analysis[tiab] OR decision analyst[tiab] OR decision analysts[tiab] OR decision analytic[tiab] OR decision analytical[tiab] OR decision analytics[tiab]) OR (decision model[tiab] OR decision modelers[tiab] OR decision modeling[tiab] OR decision modelling[tiab] OR decision models[tiab])).

Studies obtained from the database search strategy will be managed using EndNote X8 (Clarivate Analytics, Philadelphia) to identify and eliminate studies in duplicate.

### 2.3 Selection of articles

The selection of articles will be carried out in 2 stages. First, 2 independent reviewers will examine titles and abstracts. At this stage, all studies that do not adequately meet the inclusion criteria will be excluded. Second, 2 reviewers will independently carry out the integral reading and analysis of the articles based on the eligibility criteria. Doubts or discrepancies between the 2 reviewers, both in the first and second phases, will be resolved by consensus. In the case of persistence of doubt following joint evaluation, a third reviewer (senior reviewer) will be consulted. The following inclusion and exclusion criteria will be considered:

#### 2.3.1 Inclusion criteria

Inclusion criteria are: studies published in English, German, Spanish, or Portuguese language; full economic evaluation studies (cost-effectiveness, cost-utility, cost-benefit or cost-minimization) conducted throughout a clinical trial or using decision models; studies evaluating patients diagnosed or suspected of lung cancer with indication for mediastinal stage of lung cancer.

#### 2.3.2 Exclusion criteria

Exclusion criteria are: studies in which the use of EBUS and mediastinal lung cancer staging were not the focus of the evaluation; summaries published in annals of congress, editorials, letters or review articles; other economic studies that are not full economic evaluations (partial economic evaluation studies): budget impact analysis, disease cost, cost analysis studies.

### 2.4 Data extraction

Data extraction will be performed by 2 reviewers. Initial extraction will be checked and complemented as needed by a senior reviewer. The electronic form for data extraction will be constructed based on the following information: study identification (author, year, name of the periodical, country of study); type of study (cost-effectiveness, cost-utility, cost-minimization, or cost-benefit analysis); study design (trial or model based studies); population studied (characteristics of the population, number of patients, age); study perspective (society, health system, private sector, public hospital, private hospital); time horizon; intervention (EBUS-TBNA, EBUS and endoscopic ultrasound-guided TBNA); comparators (mediastinoscopy, blind-TBNA, PET-computer tomography, chest computer tomography); measures of Effectiveness; data source (systematic review and meta-analysis, controlled clinical trial, observational studies, unpublished primary data, expert opinion, assumption, other); costs (types of costs used, items included in costs, data source for costing, year in which costs were accounted for, currency unit used, inflation rate, total cost of intervention, total costs of strategies compared, discounting); modeling (decision model used, probabilities used in the model for occurrence of events and source, software, model assumptions, validation of the model); outcomes (quality adjusted life years, cost per life year gained, incremental cost effectiveness ratio, cost-benefit or cost-minimization result); sensitivity analysis (types of sensitivity analyzes performed, variables tested); cost-effectiveness threshold adopted in the study country; conclusions; other general and relevant characteristics pointed out by the authors.

### 2.5 Assessment of risk of bias

The quality assessment will be performed independently by 2 reviewers. The proposed tool to conduct the evaluation is the Consolidated Health Economic Evaluation Reporting Standards (CHEERS).^[11]^ The items to be evaluated by CHEERS are subdivided into 6 main categories: title and abstract; introduction; methods; results; discussion; and another. From these categories is contained a checklist of 24 items. The quality of study reports will be assessed against the 24 checkpoints. It will be symbolized as (√) each point that was fulfilled, symbolized as (≠) each point partially fulfilled and symbolized as (X) each point that was not attended to. For a better visual identification of the quality analysis in the table that will be presented, the fulfilled items will be painted green, the partially fulfilled in yellow, and those not attended in red. If the checkpoint does not apply to the study in question, it will not be used in the quality assessment of the test (symbolized as n.a.) and left blank.

### 2.6 Data synthesis

The data synthesis and analysis plan will be presented in a descriptive way, with an approach to the main outcomes of the selected studies, identification, and discussion of the factors that influenced the results of the economic evaluation studies on the use of the EBUS-TBNA technique versus the surgical mediastinoscopy technique for the mediastinal staging of lung cancer. A table will be assembled to synthesize the most important information on characteristics and outcomes of the identified studies.

### 2.7 Risks and ethical considerations

There are no inherent risks to the study, as it is a systematic review of published literature. No ethical approval is needed for this systematic review, as it is a literature-based study.

## 3 Discussion

This systematic review will provide a broad and detailed summary of current evidence regarding full economic evaluations of the use of EBUS-TBNA for the mediastinal staging of lung cancer. The results of this review will be disseminated through peer-reviewed publications and conference publications. Thus, it may possibly influence a more cost-effective mediastinal staging approach for patients with lung cancer around the world and help health decision makers to decide whether the EBUS-TBNA technique should be incorporated into their health systems and the best way to do so.

## Author contributions

**Conceptualization:** João Pedro Steinhauser Motta.

**Data curation:** Caroliny Samary Lobato.

**Formal analysis:** João Pedro Steinhauser Motta, Ricardo E. Steffen.

**Methodology:** Vanessa Souza Mendonça, Ricardo E. Steffen.

**Project administration:** José Roberto Lapa e Silva.

**Supervision:** José Roberto Lapa e Silva.

**Writing – original draft:** João Pedro Steinhauser Motta.

**Writing – review & editing:** José Roberto Lapa e Silva, Ricardo E. Steffen.
